# Improved Perioperative Risk Education Through the Use of an Interactive Online Anaesthesia Education Tool (iPREDICT): A Prospective, Randomised Controlled Single-Centre Clinical Trial

**DOI:** 10.3390/jcm14093131

**Published:** 2025-04-30

**Authors:** Heidi Ehrentraut, Alma Puskarevic, Andrea Kunsorg, Izdar Abulizi, Andreas Mayr, Milan Jung, Maximilian Schillings, Caroline Temme, Annika Pütz, Mark Coburn, Maria Wittmann

**Affiliations:** 1 Department of Anaesthesiology and Intensive Care Medicine, University Hospital Bonn, 53127 Bonn, Germany; alma.puskarevic@ukbonn.de (A.P.); andrea.kunsorg@ukbonn.de (A.K.); s4maschi@uni-bonn.de (M.S.); s4catemm@uni-bonn.de (C.T.); annika.puetz@ukbonn.de (A.P.); mark.coburn@ukbonn.de (M.C.); maria.wittmann@ukbonn.de (M.W.); 2 Department of Medical Biometry and Statistics, University of Marburg, 35037 Marburg, Germanyandreas.mayr@uni-marburg.de (A.M.)

**Keywords:** preoperative care, preoperative anaesthetic assessment, patient education, anaesthesia risks, interactive, online

## Abstract

**Background/Objectives**: Involving patients in the preoperative anaesthetic assessment (PAA) process can improve their understanding of risks and contribute to better postoperative recovery and outcomes. The iPREDICT study aims to investigate the feasibility of using an interactive consultation tool (ICT) to improve patient awareness of anaesthesia-related risks. **Methods**: This prospective, single-centre, randomised, placebo-controlled clinical study included patients scheduled for elective surgery under general or combined general and regional anaesthesia. Participants were randomly assigned to online anaesthesia risk education in the ICT group (intervention) or a control group that watched a video without anaesthetic risk content. Both groups received a face-to-face PAA and were assessed about anaesthetic risk knowledge after PAA and two days later. **Results**: A total of 373 participants were randomised, and 315 completed the assigned online module prior to their PAA. The proportion of male participants was higher (>60%). Most participants already had prior anaesthesia experience. After PAA, 243 patients completed the first risk recall #1 questionnaire, with the ICT group identifying significantly more correct risks than the control group (median 13.0 vs. 11.0, *p* < 0.05). In risk recall #2, conducted two days after the PAA, knowledge retention remained stable in the control group, while the experimental group showed further improvement (median 14.0 vs. 13.0, *p* < 0.05). **Conclusions**: Using the ICT significantly improved patients’ knowledge of anaesthesia-related risks. These results suggest that interactive patient education tools are a feasible and effective way to improve patients’ understanding of perioperative anaesthesia risks, potentially contributing to better outcomes, which needs to be addressed in future projects.

## 1. Introduction

Preoperative anaesthetic assessment (PAA) plays a crucial role in preparing patients before surgery by addressing their concerns, providing general information about anaesthesia, obtaining specific information relating to their condition, and obtaining informed consent [1]. An essential component of this process is patient empowerment, which comprises education and patient involvement in decisions related to their anaesthesia care [2,3]. Promoting patient involvement and informed decision-making is crucial for patient safety. Enhancing patients’ strengths and strategies in coping with health situations leads to improved health outcomes, optimised care and overall well-being [4]. Empowerment derives from patient motivation and learning, collaboration and mutual trust between patient and healthcare professional [5].

In the context of the COVID-19 pandemic and the increasing burden on the healthcare system, the use of telemedicine and online patient education comes to the fore [6]. Personnel and economic resources of the hospital organisation may benefit from an interactive risk education tool, which improves the PAA process by reducing time consumption and streamlining workflows. Furthermore, supplementary media-based interventions and telemedicine can improve patients’ preoperative knowledge, increase patient empowerment and satisfaction and reduce anxiety, perioperative risks and length of stay [7,8,9,10,11,12]. In routine clinical practice, many factors may compromise the result of the PAA [9]. From a patient’s perspective, communication barriers, lack of knowledge or willingness to provide complete and accurate testimony of the current situation leads to an incomplete or wrong picture of the patient’s health situation [13,14,15]. In these often-stressful situations, the patient may not memorise important aspects (e.g., taking or discontinuing medication or following, e.g., fasting recommendations), or the anaesthesiologist may not structurally query certain parameters due to time constraints or communication problems [15,16,17].

Study results have shown to some extent that additional digital education increases patient satisfaction and the effectiveness of education [7]. Involving the patient in the preoperative decision-making process is key during anaesthesia education and has a positive influence on the patient’s compliance with the anaesthetist’s preoperative orders, as well as the patient outcome [3]. This is also true with regard to the fact that discussions with the anaesthetist are frequently held in exceptional situations characterised by preoperative anxiety or distress [18]. In the field of anaesthesiology education, there is currently limited evidence regarding the integration of risk education tools in the PAA process [19]. Further research is needed to evaluate and implement an educational tool during the PAA. Such a tool could improve patient’s understanding of risks, promote active participation in risk avoidance and improve postoperative recovery and outcome. Additionally, patient autonomy and comfort could also benefit from an interactive consultation tool in the future. For low-risk patients, a remote consent process could eliminate the need for non-essential hospital investigations [12].

The aim of this randomised placebo-controlled clinical study is, therefore, to investigate whether the preoperative use of an interactive consultation tool (ICT) is a viable way to improve patients’ knowledge and awareness of the risks associated with anaesthesia. In addition, this study investigated preoperative anxiety, need for information and patient satisfaction.

## 2. Materials and Methods

### 2.1. Study Design

**Figure 1:**
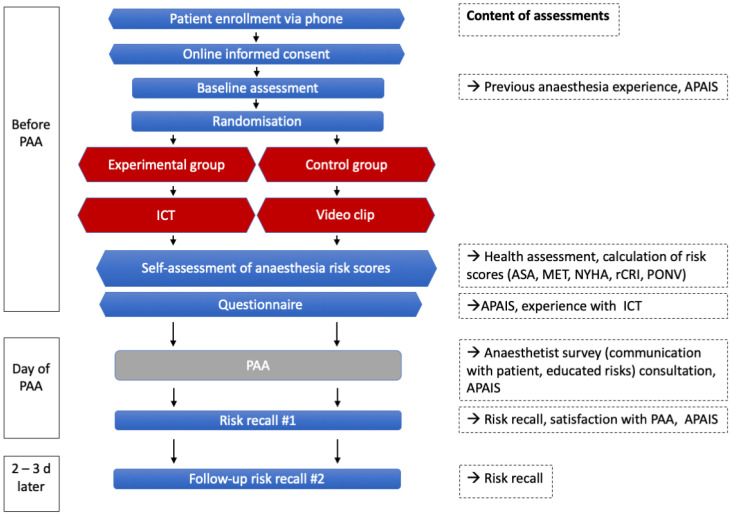
Flow chart of the iPREDICT study. Amsterdam Preoperative Anxiety and Information Scale (APAIS), American Society of Anaesthesiologists (ASA), interactive consultation tool (ICT), metabolic equivalent of task (MET), New York Heart Association (NYHA), preoperative anaesthetic assessment (PAA), postoperative nausea and vomiting (PONV), Revised Cardiac Risk Index (rCRI).

The iPREDICT study was a prospective, randomised, placebo-controlled clinical study conducted at the University Hospital Bonn, Germany. Recruitment lasted 17 months, from September 2023 to January 2025. Patients scheduled for PAA were enrolled and participated in online anaesthesia risk education via ICT (intervention group) or watched a video containing patient safety-related general hospital information without anaesthetic risk content (control group), prior to a face-to-face PAA (see Figure 1 for study flow chart). The Ethics Committee of the Medical Faculty of the University Hospital Bonn approved the trial protocol on 26 September 2022 (no. 338/22). The study is registered in the German Clinical Trials Register (https://drks.de/search/en/trial/DRKS00032514 (accessed on 10 March 2025)).

### 2.2. Patient Enrolment

All patients aged 18 years and older, who were scheduled for elective surgery under general anaesthesia or combined regional and general anaesthesia, were eligible for inclusion. Upon scheduling of their appointment in the preoperative anaesthesia outpatient clinic they were contacted by phone. Exclusion criteria included emergency surgery, insufficient knowledge of German, inability to give informed consent or participate in the study due to lack of internet access or email address. The entire spectrum of surgical procedures was carried out at our facility, but not all surgical departments made use of advance appointments via the anaesthesia outpatient clinic. If patients were unwilling to participate, their reasons were recorded.

Patients were also informed that they could complete the education process independently or with the assistance of family members, to address potential issues or insecurities related to media literacy.

### 2.3. Data Collection and Security

After verbally consenting to study participation, the patient’s identification number and their email address were entered on a secure online tool, and patients received a link to an online informed consent form (ICF) as well as the detailed study information. All pseudonymised questionnaire results were processed via the General Data Protection Regulation conform software, developed by Medify (Amsterdam, The Netherlands). The organisation’s systems are ISO/IEC 27001-certified and aligned with the control recommendations outlined in ISO/IEC 27002 [20,21]. The audio–visual ICT content was hosted within the Medify software framework. Randomisation and allocation were performed within the software using the Linux kernel’s ChaCha20-based cryptographically secure pseudo-random number generator (CSPRNG) [22].

### 2.4. Randomisation/Baseline

After enrolment and providing digital consent, baseline data were assessed, and participants were randomly assigned to the control or ICT intervention group in a 1:1 ratio. During baseline data collection, sociodemographic data (gender, age), frequency of previous anaesthesia experiences, patients’ anxiety levels and need for information in the preoperative phase were captured, using a six-item questionnaire, the Amsterdam Preoperative Anxiety and Information Scale (APAIS) [23].

### 2.5. Intervention

The ICT intervention provided general information covering the anaesthesia outpatient clinic, procedural steps on the day of the anaesthetic, induction, the operating theatre and recovery room, as well as associated risks (Table S1A) and precautions for risk reduction. The ICT contained videos that were recorded at the University Hospital Bonn, containing both voice-overs and readable text boxes.

Following the ICT risk education and information session, patients were asked several questions about their experience with the ICT. They rated the clarity, completeness, and informativeness of the session, the adequacy of its duration, and whether it alleviated their anaesthesia-related concerns.

The control group watched a video about general hospital and patient safety information without anaesthetic risk content (control group). Finally, the APAIS score was recorded.

### 2.6. Risk Recall #1/PAA Appointment

On PAA day, patients provided written informed consent. Immediately after their face-to-face consultation, the patient and anaesthesiologist completed a questionnaire on time spent and satisfaction with the consultation process. Anaesthesiologists stated on a 4-point Likert scale whether patients brought along required medical documents, provided relevant information, asked many questions or refused anaesthesia education. Patients rated whether the anaesthetists took enough time to provide information and answer questions or conveyed the information in an understandable way (4-point Likert scale). They indicated whether waiting time for the PAA was acceptable and whether the appointment relieved their fear and worries. Both groups were assessed immediately after the face-to-face consultation and tested on the previously discussed perioperative risks. In addition to the actual possible risks, five incorrect answers were included (Table S1B). Patients rated the risks using four levels of awareness, i.e., certainly known, perhaps known, rather not known, not known at all. In the evaluation, these categories were summarised as known or unknown. Risk knowledge score was calculated as the number of correctly recognised risks. Furthermore, the APAIS score was surveyed, and participants were asked which consultation method they would prefer in the future. 

### 2.7. Risk Recall #2/Two Days After Consultation Day

Two days after their consultation visit, all patients received an online questionnaire with the same risk items as before, enquiring about the memorised anaesthesia risks and ability to recognise incorrect answers.

### 2.8. Outcome Parameters

The primary efficacy endpoint was the assessment of the patient’s perioperative risk knowledge score after the PAA appointment. The secondary endpoints measured were differences in face-to-face interview duration between experimental and control group (time efficiency), the satisfaction with the consultation process (experimental vs. control, patient and anaesthetist), and between short- and long-term risk recall scores (risk recall #1 vs. risk recall #2, experimental vs. control).

### 2.9. Sample Size

The sample size was determined based on a power calculation for the primary efficacy endpoint. Using Fisher’s exact test (comparing two-sample proportions) with a two-sided significance level of 0.05, we assumed an expected difference in the proportion of correctly recalled risk from 57.1% to 75.7% based on the results in the literature [24]. To achieve a power of at least 80% based on these assumptions, at least *n* = 110 patients should be analysed per group. Adjusting for potential confounders via logistic regression was assumed to further increase the power.

### 2.10. Statistical Analysis

The statistical programming environment R version 4.4.0 (Foundation for Statistical Computing, Vienna, Austria) was used for statistical analysis. Outcome parameters are presented as median [Q1, Q3] for continuous variables and as numbers and percentages (%, missing values excluded) for nominal variables. To test for statistical differences between both groups, unless specified otherwise, we computed the nonparametric Wilcoxon rank sum test for continuous or ordered variables, and Fisher’s exact test for nominal variables; corresponding nonparametric tests for paired samples were used for differences in the control/experimental group from PAA visit to follow-up risk recall. To evaluate the effect of different factors on risk recall performance, multivariate linear regressions were estimated with the number of correctly recognised risks as outcome variables, controlling for group affiliation. The effect of risk education by anaesthesiologist on the individual risk factor was assessed by computing individual linear regressions with recognition of the individual risks (on a Likert scale of 1–4) as the outcome variable, controlling for group affiliation.

A significance level of 0.05 was used for all statistical tests without adjustment for multiple testing due to the exploratory nature of the trial.

## 3. Results

**Figure 2:**
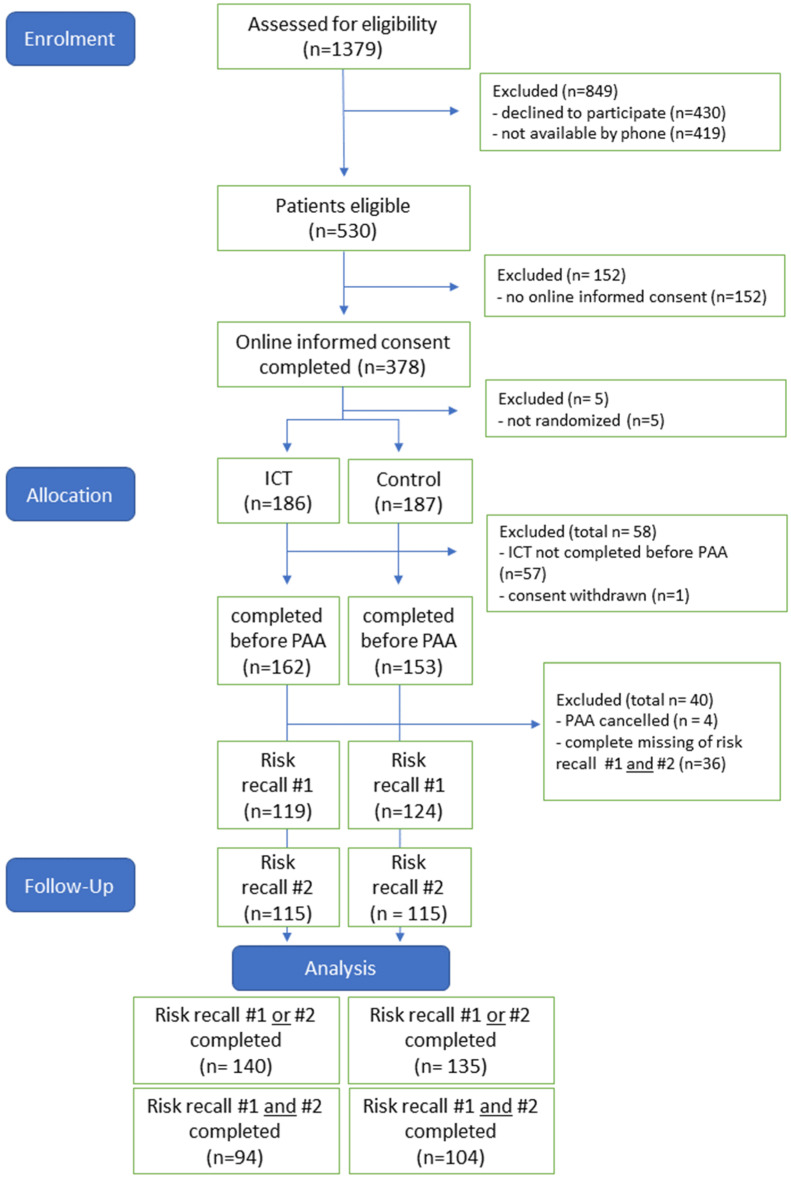
Consort diagram.

During the iPREDICT trial, 960 patients were successfully contacted by telephone (Figure 2). The reasons for refusal of participation (*n* = 430) were noted (Table S2), and 530 patients verbally agreed to study participation. The cluster not willing to participate in the study was older and contained a higher proportion of females (Table S3).

The first contact with the eligible patients had to be made by telephone, and randomisation was only permitted after online consent had been given. A proportion of 28% of the 530 enrolled patients did not complete the online informed consent. Protocol adherence by the patients was mediocre, even though email invitations at each step of the study were followed by email reminders. Upon digital study consent being given, 373 participants were randomised. A total of 315 patients (60% of 530 enrolled patients) completed the assigned online module in time, i.e., prior to their PAA appointment. If data for the efficacy endpoint (knowledge about perioperative risks) were collected on either PAA day or the follow-up risk recall visit, patients were included in data evaluation (*n* = 275 (52% of 530)). Only 39% completed both surveys.

### 3.1. Baseline Characteristics

No differences occurred between control and experimental ICT group regarding age, gender, ASA score (Table 1), and previous anaesthesia experience (Table S4). The demographic analysis showed that the reduction in cohort size due to dropouts did not significantly alter the age and gender distribution, supporting the validity of the findings. The overall percentage of male participants was higher (>60%). This can be partly explained by the most frequently performed surgical procedures (Table 1). The proportion of male patients was greater for urological and maxillofacial interventions, accounting for 69% of all urological and head/neck procedures in this cohort.

More than 80% of the patients had undergone previous anaesthesia (Table 1). For more than 50% of the study population, two years or less had passed since the last surgical intervention with general anaesthesia (Table S4).

Patients had a median outward and return journey of 56 km, i.e., 31% had to drive a total of at least 100 km for their appointment in the outpatient clinic.

### 3.2. Anaesthesia Risk Recall

After completion of the PAA visit 243 patients answered the risk recall questionnaire #1 in the anaesthetic outpatient ambulance. Participants of the ICT group identified a median number of 13.0 [Q1, Q3: 10.0, 15.0] out of 15 correct risks, and performed significantly better than those of the control group (median 11.0 [Q1, Q3: 8.0,14.0], *p* < 0.05), (Table 2). The groups did not differ in naming false positive risks (1.0 [Q1, Q3: 0.0, 2.0]). *n* = 230 patients filled out the online follow-up risk recall #2 questionnaire. The previous significant group difference in favour of the ICT group was confirmed (median 14.0 [Q1, Q3: 12.0, 15.0] vs. control: 11.0 [9.0, 14.0], *p* < 0.05), indicating that the number of correctly identified risks remained stable in the control group, whereas the experimental group attained even better results in this survey (median 14.0 [Q1, Q3: 12.0,15.0] vs. 13.0 [Q1, Q3: 10.0, 15.0], *p* < 0.05), (Table S5).

Overall, nausea, allergic reactions and a sore throat were the perioperative risks best known among all participants on the PAA day (Table S6A). Attendance for the online education significantly improved the identification of teeth damage, postoperative delirium, serious events (e.g., heart or brain issues, death), pneumonia and hyperthermia. During the follow-up risk recall, the ICT group recognised 12 out of 15 perioperative risk items with a significantly higher rate than the control group (Table S6A).

Immediately after consultation, test results from the control group reflected the working memory, which is responsible for the temporary storage and processing of information [25]. Since completion of the online education, 2 days [Q1, Q3: 1.0, 4.0] had passed until risk recall #1 on PAA day in the ICT group, which led to the retrieval of long-term memories. A total of 86% of the patients from the experimental group passed this first risk recall test at least 1 day after ICT completion. In risk recall #2, long-term memory, which stores information over a longer period, was examined in both groups [26]. A median of 3 days [Q1, Q3: 2.0, 4.0] had elapsed in both groups until follow-up risk recall completion. The risk recall #2 questionnaire was sent out 2 days after PAA. Overall, 174 patients completed the survey on the second or third day after the PAA, as initially intended, 53 patients finished the test 4–9 days after PAA, and three patients filled in the test more than 90 days later.

We inserted wrong answers on purpose to find out whether patients deliberately ticked what they had read. In the anaesthetic outpatient ambulance, twelve patients (six per group) checked all 20 answers as “certainly known”, six patients (three per group) checked all answers as “not known at all”. During online follow-up risk recall, this number reduced and only three patients (control group: one, ICT group: two) ticked all answers, and only two patients from the control group recognised no risk at all. This indicates that the test response in an unsettled hospital environment produces less reliable results.

### 3.3. Anaesthesiologist—Survey and Risk Explanation

During the PAA, anaesthetists informed the patients about nine of the risks that were also mentioned in the patient’s risk recall survey. No difference occurred between the control and ICT group (control: median 9.0 [Q1, Q3: 6.0, 14.0]; experimental: 9.0 [Q1, Q3: 6.0, 15.0]). The five risks most often discussed (63–77%) were allergic reactions, nausea, damage to the teeth, sore throat and serious life-threatening events. The potential risks least often (<37%) considered by the anaesthetist were hyperthermia, thrombosis/embolism, awareness, laryngospasm, blood loss and postoperative delirium (Table S6B).

Partially in line with this, multivariate linear regression analysis revealed that verbal communication about the occurrence of allergic reactions and tooth damage significantly enhanced the recall of these risks right after the PAA (Table S6C). Whether postoperative delirium (POD) or the risk of a sore throat was verbally communicated had a significant effect on the patient’s follow-up risk recall result. However, the overall effect size weakened between the two time points.

The median time required for each PAA did not vary between the groups (30.0 min [Q1, Q3: 30.0, 30.0]). The anaesthesiologists rated the quality of conversation and communication equally in both groups (Table S7). However, they reported differences when bringing medical documents. Significantly more patients in the experimental group brought medication plans (57 vs. 36 patients (control), *p* < 0.05) and GP letters (11 vs. 2 patients (control), *p* < 0.05) with them (Table S7). Fewer patients from the ICT group rejected a detailed risk education (31% vs. 43%, not significant).

### 3.4. Preoperative Anxiety and Demand for Qualified Information

We inquired about the demand for qualified information about the upcoming anaesthesia and procedure, as well as the presence of preoperative anxiety, at three different timepoints. A pronounced anxiety could not be determined, and hence, the overall anaesthesia related anxiety was low. The group comparison revealed a significant difference at baseline, prior to randomisation with slightly higher anaesthesia anxiety scores for patients assigned to the ICT group (*p* < 0.05) (Table S8). After ICT completion and anaesthesiologist consultation, the values between the groups did not differ significantly. The other items of the APAIS score showed no significant differences. Patients in both groups reported a somewhat more pronounced surgery-related anxiety. In line with this, the demand for surgical procedure-related information exceeded the need for anaesthesia-related details. It was noticeable that both the control intervention, the experimental intervention, as well as the interview, improved the overall anxiety score. In line with this, most patients from the control (56%) and ICT groups (69%) would choose a combined anaesthesia education, including online education at home as well as personal explanations from a physician (Table S9).

### 3.5. Multivariate Regression Analysis of Relevant Variables for the Risk Recall Performance

To gain a better impression into which variables are favourable for risk recall performance, four factors were included in a multivariate regression analysis (Table 3). None of the predictors met the level of significance on PAA day. We observed a positive effect size of participation in the ICT group (*p* > 0.05). During follow-up risk recall, ICT group affiliation significantly affected the risk recall result with an estimated effect size of 2.21 (*p* < 0.001), indicating an increase in the expected number of correctly recognised risks at risk recall #2 from the use of ICT. An influence of previous anaesthesia numbers or the time point of ICT completion could not be confirmed. We did, however, find that the need for anaesthesia information expressed in the APAIS baseline survey increases the number of correctly recognised risks (estimated effect size 0.55 for each step on the 5-point Likert scale: 1—very low demand to 5—extremely high demand).

### 3.6. Patient Satisfaction with Anaesthesia Education

Asked about their experience with the PAA, patients overall regarded their contact with the anaesthesiologist as satisfactory (Table S10). The waiting time received mixed ratings. Patients from the experimental group were also asked about their ICT experience after ICT completion and gave it good reviews in terms of content and timely effort (Table S11). When it came to reducing worries and fears, patients benefited more from the face-to-face conversation than from the online tool.

## 4. Discussion

We collected data about risk knowledge retention in 275 patients and observed that the completion of an online anaesthesia consultation module significantly improved the participants’ performance. The ICT had a stronger effect on recall scores than previous anaesthesia experiences or risk disclosure outpatient anaesthesia appointments. In addition, the patients’ individual need for anaesthesia-related information was an independent factor for test performance.

To the best of our knowledge this is the first trial investigating the feasibility of an interactive consultation module in Germany. Age had a significant influence on the patient’s willingness to participate in this trial. The largest proportion did not disclose their reasons for rejection. 45% of the contacted persons refused participation. 17% of those who rejected participation gave testimony that the reason for the obstruction was the lacking ability to use the digital application. As assumed, those who named these criteria were older. However, digital media competence will most likely improve further in the years to come [27,28], and so will the legal framework conditions for the digital sharing of health data. Nevertheless, it must be considered that health literacy, as a social determinant of health, is a challenge. Vulnerable individuals, who might profit the most from increased health literacy and digital health literacy, have the worst chance of gaining access to it [29,30].

Satisfaction with the face-to-face PAA and the ICT was high, and most patients stated that they prefer a combination of both in the future. Building a relationship to the healthcare professionals and acquiring trust requires personal contact. However, audio–visual content allows for better knowledge transfer of medical information. The participants did not compare the two methods to each other and evaluated them right after completion of the respective methods. Incorporating the patients’ opinion during the future development of online education tools and offering the opportunity to obtain individualised content may further increase their acceptance. So far, only some patients took advantage of the offer, to involve their relatives in the process and to look at the information together. However, 19% reported that they had viewed the content several times, an option that has many advantages. As long as the visit to the outpatient clinic was not accompanied, it is more difficult to recapitulate the information heard. Still, alternatives must be considered since there will never be a full elimination of technological barriers due to various reasons.

The main aim of our study was to investigate how well patients understood anaesthesia-related information at two different time points, reflecting short- and long-term storage. In line with previous studies, supplementation of the face-to-face consultation with our digital intervention increased the information gain, even though study conditions varied greatly [19,24,31,32,33]. Other working groups used questionnaires with less items or complex multiple-choice tests, querying knowledge about the anaesthetic procedure. Some used brochures as control intervention and many did not test long-term knowledge retention, probably due to practicability and data protection reasons. In our study, this later time point proved to be particularly significant, clearly highlighting group differences. The improved learning effect in the ICT group can be attributed to their ability to connect the visual ICT content with their existing knowledge and experiences. The conversation with the anaesthetist reinforced and expanded this understanding. The first risk recall test reactivated the knowledge, augmenting it through test-enhanced learning. This multi-stage process of learning and repetition led to even stronger knowledge retrieval in the final risk recall test [34].

Our knowledge retention test only covered the topic of anaesthesia-related risks. Though after taking the anaesthesiologist survey into consideration, we observed that more patients from the experimental group brought in relevant medical records compared to the control group, concluding that other important aspects of the education have been understood as well. Furthermore, a mixed design set-up, made of a combination of pre-hospital digital risk education with the preformed questions regarding the topic, could not only strengthen trust, but also empower patients in their self-efficacy through test-enhanced learning effects.

Half of our study cohort expected a low-risk intervention, though only a small proportion, 14%, were ASA 1 patients. The increasing population of elderly, multi-morbid patients presents additional challenges. These patients often require longer consultation times due to complex medical histories, polypharmacy and the need for more detailed risk-benefit discussions. However, demographic change and physician shortages mean that the number of available physicians cannot be increased to the same extent. This discrepancy could lead to reduced patient safety and patients’ outcomes if no other solutions are implemented. Notably, 50% of this study cohort had experienced previous surgery within the last two years. For a notable amount of those cases, face-to-face consultation may be substituted by equivalent, reasonable and safe digital consultation, which relieves infrastructure and also saves time and CO_2_, in the future [35].

### Limitations

We did not incorporate a reference risk knowledge assessment of patients, who had not participated in any kind of in-person or online anaesthesia consultation. Therefore, our study does not consider the different examination conditions of a risk survey performed in the hospital or at home. The first risk assessment took place in a louder and more turbulent hospital environment than the second assessment, the exact time of which was determined by the patients themselves. Still, we can conclude from our results that in subsequent studies on patients undergoing elective surgeries, a risk survey at a later date is sufficient and more meaningful.

The anaesthetists were not aware of the patient’s group allocation; however, full blinding was not possible or intended. Still, we cannot exclude bias, and we did not directly measure the extent to which the treatment influenced the course of conversation during PAA.

## 5. Conclusions

Incorporating supplementary online consent tools in the perioperative clinical practice offers significant advantages for patient empowerment and decision-making. Furthermore, it provides advantages in terms of time efficiency and in CO_2_ savings. With an underlying legal framework for selected low-risk patients, personal appointments in the anaesthesia outpatient clinic may be saved in the future, and this could lead to a reduction in personal capacity in favour of patients at risk.

## Figures and Tables

**Table 1:** Baseline characteristics—all patients with at least one completed risk recall assessment.

	Control (n = 135)	Experimental (n = 140)	*p*-Value
Age (y)			
Median [Q1, Q3]	58.0 [46.0, 67.0]	56.0 [41.8, 67.0]	0.36
Gender			
Male	95 (70%)	88 (63%)	0.203
Female	40 (30%)	52 (37%)	
Distance from home to UKB (km)			
Median [Q1, Q3]	26.0 [14.0, 61.0]	29.0 [16.0, 51.5]	0.835
Surgery risk			
Cardiosurgical	3 (2%)	4 (3%)	0.101
High	0 (0%)	6 (4%)	
Intermediate	59 (46%)	58 (43%)	
Low	67 (52%)	66 (49%)	
Missing	6	6	
Previous anaesthesia			
Yes	115 (92%)	115 (90%)	0.663
No	10 (8%)	13 (10%)	
Missing	10	12	
ASA score (anaesthesiologist)			
1	22 (17%)	19 (14%)	0.404
2	87 (65%)	91 (65%)	
3	22 (17%)	30 (21%)	
4	2 (2%)	0 (0%)	
Missing	2		
Surgical specialty			
Urology and kidney	58 (45%)	64 (48%)	0.819
Head and neck	51 (40%)	48 (36%)	
Orthopaedics	5 (4%)	7 (5%)	
Vascular	2 (2%)	5 (4%)	
Heart	3 (2%)	4 (3%)	
Lower gastrointestinal tract	4 (3%)	1 (1%)	
Plastic/dermatologic	1 (1%)	2 (1%)	
Thorax (lung and other)	2 (2%)	1 (1%)	
Hepato-biliary	1 (1%)	1 (1%)	
Gynaecology and Obstetrics	0 (0%)	1 (1%)	
Upper gastrointestinal tract	1 (1%)	0 (0%)	
Other	1 (1%)	0 (0%)	
Missing	6	6	
If surgery performed: Type of anaesthesia			
General anaesthesia	122 (95%)	129 (96%)	0.652
Combined general and regional anaesthesia	4 (3%)	4 (3%)	
Regional anaesthesia	3 (2%)	1 (1%)	
Missing ^1^	6	6	

^1^ Missing values include cases of analgosedation or local anaesthesia.

**Table 2:** Number of recognised risks. Comparison between control and experimental group (A) on PAA day. (B) upon follow-up risk recall two days after PAA.

A. PAA Visit—Risk Recall #1.	Control (n = 124)	Experimental (n = 119)	*p*-Value
Number of correctly recognised risks			0.0446
Median [Q1, Q3]	11.0 [8.0, 14.0]	13.0 [10.0, 15.0]	
Mean (±SD)	10.1 (±4.83)	11.1 (±4.68)	
Number of false positive risks			0.97
Median [Q1, Q3]	1.0 [0.0, 2.0]	1.0 [0.0, 2.0]	
Mean (±SD)	1.35 (±1.61)	1.31 (±1.56)	
**B. Follow-up—Risk Recall #2**	**Control** **(n = 115)**	**Experimental** **(n = 115)**	
Number of correctly recognised risks			0.0248
Median [Q1, Q3]	11.0 [9.0, 14.0]	14.0 [12.0, 15.0]	
Mean (±SD)	10.7 (±4.17)	12.7 (±2.84)	
Number of false positive risks			0.36
Median [Q1, Q3]	1.0 [0.0, 2.0]	1.0 [0.0, 3.0]	
Mean (±SD)	1.38 (±1.49)	1.56 (±1.55)	

**Table 3:** Multivariate regression analysis. Effect of independent variables group affiliation, previous anaesthesia, demand for information about anaesthesia (APAIS baseline), and time point of ICT completion on dependent variable knowledge retention. (A) after premedication visit, (B) 2–3 d follow-up risk recall.

A. PAA Visit Risk Recall#1				B. Follow-Up Risk Recall#2			
Predictors	Estimates	CI	*p*	Predictors	Estimates	CI	*p*
(Intercept)	9.60	7.43–11.77	<0.001	(Intercept)	9.33	7.73–10.93	<0.001
Group (ref. = control)	1.05	−0.21–2.32	0.103	Group (ref. = control)	2.21	1.24–3.18	<0.001
Number previous anaesthesias ^1^	0.05	−0.07–0.17	0.442	Number of previous anaesthesias ^1^	−0.03	−0.12–0.05	0.448
Information demand ^1^	0.20	−0.45–0.84	0.553	Information demand ^1^	0.55	0.06–1.04	0.027
Days from ICT to PAA visit ^1^	−0.11	−0.26–0.03	0.122	Days from ICT to risk recall ^1^	−0.01	−0.05–0.03	0.628
Observations	221			Observations	211		
R^2^/R^2^ adjusted	0.027/0.009			R^2^/R^2^ adjusted	0.117/0.100		

^1^ reference value = null.

## Data Availability

The datasets presented in this article are not readily available because the data are part of an ongoing sub-study analysis. Requests to access the datasets should be directed to the corresponding author.

## Supplementary Materials

The following supporting information can be downloaded at: https://www.mdpi.com/article/10.3390/jcm14093131/s1. Table S1. List of perioperative risk items with (A) 15 correct and (B) 5 false risks. Table S2. Reasons for not consenting in trial participation. Table S3. Age and gender distribution in patients providing consent or not wishing to participate. Table S4. Details about previous anaesthesia experience. Table S5. Comparison of knowledge retention at PAA visit (risk recall #1) vs. follow-up risk recall #2 (patients with both primary endpoints). Table S6. Overview of recognised and explained anaesthesia risks. A. Numbers (%) of correct answers in risk recall #1 and #2 (correct risks and incorrect answers identified). B. Explained risks by anaesthesiologist. C. Multivariate linear regression analysis. Estimated effect of risk education by anaesthesiologist on dependent variable single risk identification on a Likert scale (1–4), controlled for effect of group affiliation. Table S7. Anaesthesiologist survey. A. Communication with patient. B. Documents provided to anaesthesiologist by the patient during PAA visit. Table S8. Amsterdam Perioperative Anxiety Score (APAIS). Table S9. Choice education mode. Table S10. Patient satisfaction with PAA appointment. Table S11. Satisfaction with online anaesthesia education.

## Abbreviations

The following abbreviations are used in this manuscript:

ICT	Interactive consultation tool
PAA	Preoperative anaesthetic assessment
Q1, Q3	Quartile 1 and quartile 3 define the lower and upper limits of the interquartile range
