# Supplementary material for: Improved Perioperative Risk Education Through the Use of an Interactive Online Anaesthesia Education Tool (iPREDICT): A Prospective, Randomised Controlled Single-Centre Clinical Trial
Source: J Clin Med. 2025 Apr 30;14(9):3131. doi: 10.3390/jcm14093131 (PMC12072581; doi:10.3390/jcm14093131)
Supplement: Supplementary file 1 [file jcm-14-03131-s001.zip › jcm-3567107-supplementary.pdf]

Table S1. List of perioperative risk items with (A) 15 correct and (B) 5 false risks.

	**A Perioperative risks**	**B False risks**
**1**	Allergic reaction to administered medication	Hair loss
**2**	Bruising or nerve damage due to an injection or infusion and positioning damage	Stomach ulcer
**3**	Infections at puncture sites or catheters	Osteoporosis
**4**	Low blood pressure	Flatulence
**5**	Blood loss, transfusion requirements	Impaired sense of smell
**6**	Thrombosis, embolism	
**7**	Nausea and vomiting	
**8**	Pneumonia	
**9**	State of confusion, delirium	
**10**	Tooth damage	
**11**	Sore throat and hoarseness	
**12**	Cramping of the airways	
**13**	Waking up during the operation	
**14**	Life-threatening rise in body temperature	
**15**	Heart and breathing problems, brain damage and death	

Table S2. Reasons for not consenting in trial participation.

	Age (y)	Gender	
	**Median [Q1, Q3]**	**M<br>(N=240)**	**W<br>(N = 160)**
Overall (N=430)	61.0 [44.0, 70.0]	240 (56 %)	190 (44 %)
Does not wish to participate (N=134)	63.0 [42.3, 70.0]	76 (57 %)	58 (43 %)
Time constraints (N=92)	54.0 [39.3, 63.0]	60 (65 %)	32 (35 %)
No media competence (N=41)	70.0 [63.0, 76.0]	24 (59 %)	17 (41 %)
Lack of German language skills (N=31)	47.0 [38.0, 70.0]	13 (42 %)	18 (58 %)
Other limitation (such as visually impaired/blind,<br>hard of hearing /deaf) (N=23)	52.0 [41.5, 67.0]	12 (52 %)	11 (48 %)
Unable to give consent (N=16)	40.5 [34.0, 61.8]	11 (69 %)	5 (31 %)
No internet access (N=15)	75.0 [65.5, 79.0]	5 (33 %)	10 (67 %)
No email address (N=14)	66.0 [63.3, 76.8]	7 (50 %)	7 (50 %)
No general anaesthesia planned (N=12)	62.5 [55.8, 74.0]	11 (92 %)	1 (8 %)
Data protection concerns (N=1)	70.0 [70.0, 70.0]	0 (0 %)	1 (100 %)
Other reasons (N=51)	61.0 [47.0, 68.0]	21 (41 %)	30 (59 %)

Table S3. Age and gender distribution in patients providing consent or not wishing to participate.

	**Consent (N=530)**	**No consent (N=430)**	**P-value**
**Age (y)**			
Median [Q1, Q3]	56.0 [40.0, 66.0]	61.0 [44.0, 70.0]	**<0.001**
**Gender**			
m	336 (63 %)	240 (56 %)	**0.02**
w	194 (37 %)	190 (44 %)	

Table S4. Details about previous anaesthesia experience. Sub-questions only expanded if precondition was fulfilled (previous main question = YES).

	Control (N=135)	Experimental (N=140)	P-value
**1) Previous anaesthesia**			
Yes	115 (92%)	115 (90 %)	0.663
No	10 (8%)	13 (10%)	
Missing	10	12	
**a. Number of previous anaesthesias**			
Median [Q1, Q3]	4.00 [2.0, 6.0]	4.00 [2.0, 6.0]	0.83
No previous anaesthesia	10	13	
**b. Previous emergency surgery**			
Yes	22 (19 %)	25 (22 %)	0.744
No	93 (81 %)	90 (78 %)	
Missing	20	25	
**i. Number of previous emergency surgeries**			
Median [Q1, Q3]	1.00 [1.0, 1.75]	1.0 [1.0, 1.0]	0.628
Missing	113	117	
**c. Years since last general anaesthesia**			
Median [Q1, Q3]	2.00 [0, 7.0]	2.00 [0.25, 7.0]	0.949
Missing	24	30	
**ii. Last intervention was emergency surgery**			
Yes	13 (11 %)	14 (12 %)	1
No	102 (89 %)	101 (88 %)	
Missing	20	25	

Table S5. Comparison of knowledge retention at PAA visit (risk recall #1) vs. follow-up risk recall #2 (patients with both primary endpoints).

**Control group**	**PAA visit – Risk recall #1 (N=104)**	**Follow-up – Risk recall #2 (N=104)**	**P-Value**
Number of correctly recognized risks			0.254
Median [Q1, Q3]	11.0 [8.0, 14.0]	11.5 [9.0, 14.0]	
Mean (±SD)	10.3 (± 4.77)	10.8 (± 3.98)	
Number of false positive risks			0.858
Median [Q1, Q3]	1.0 [0.0, 2.0]	1.0 [0.0, 2.0]	
Mean (±SD)	1.38 (± 1.63)	1.38 (± 1.49)	

**Experimental group**	**PAA visit – Risk recall #1 (N=94)**	**Follow-up – Risk recall #2 (N=94)**	
Number of correctly recognized risks			**0.0108**
Median [Q1, Q3]	13.0 [10.0, 15.00]	14.0 [12.0, 15.0]	
Mean (±SD)	11.0 (± 4.66)	12.9 (± 2.71)	
Number of false positive risks			**0.0322**
Median [Q1, Q3]	1.0 [0.0 2.0]	1.0 [0.0, 3.0]	
Mean (±SD)	1.33 (± 1.57)	1.68 (± 1.56)	

Table S6. Overview of recognised and explained anaesthesia risks. A Numbers (%) of correct answers in risk recall #1 and #2 (correct risks and incorrect answers identified). B Explained risks by anaesthesiologist. C Multivariate linear regression analysis. Estimated effect of risk education by anaesthesiologist on dependent variable single risk identification on a likert scale (1-4), controlled for effect of group affiliation.

**A**	**PAA visit risk recall #1**			**Follow-up risk recall #2**			**B** **Explained risks by anaesthesiologist**		
Number (%) correct answers	Control (N=124)	Experimental (N=119)	P-value	Control (N=124)	Experimental (N=119)	P-value	Control (N = 135)	Experimental (N=140)	P-value
Nausea	107 (86 %)	102 (86 %)	0.617	106 (92 %)	110 (96 %)	**0.0103**	81 (73 %)	85 (77 %)	0.538
Allergic reaction	106 (85 %)	98 (82 %)	0.563	101 (88 %)	106 (92 %)	0.629	86 (77 %)	86 (77 %)	1
Sore throat	104 (84 %)	97 (82 %)	0.595	101 (88 %)	110 (96 %)	**0.0125**	83 (75 %)	78 (70 %)	0.454
Puncture injury	99 (80 %)	98 (82 %)	0.673	98 (85 %)	109 (95 %)	0.0538	52 (47 %)	54 (49 %)	0.688
Puncture infection	95 (77 %)	97 (82 %)	0.415	98 (85 %)	110 (96 %)	**0.00538**	52 (47 %)	54 (49 %)	0.79
Damage teeth	87 (70 %)	93 (78 %)	**0.00237**	82 (71 %)	99 (86 %)	**< 0.001**	82 (74 %)	84 (76 %)	0.759
Thrombosis/embolism	84 (68 %)	92 (77 %)	0.0944	91 (79 %)	101 (88 %)	**< 0.001**	26 (23 %)	29 (26 %)	0.643
Blood loss	78 (63 %)	84 (71 %)	0.136	78 (68 %)	94 (82 %)	**0.0473**	34 (31 %)	43 (39 %)	0.206
POD	78 (63 %)	90 (76 %)	**0.0187**	80 (70 %)	104 (90 %)	**< 0.001**	38 (34 %)	43 (39 %)	0.488
Hypotension	77 (62 %)	79 (66 %)	0.314	71 (62 %)	91 (79 %)	**0.0077**	67 (60 %)	68 (61 %)	0.892
Laryngospasm	77 (62 %)	80 (67 %)	0.595	77 (67 %)	90 (78 %)	**0.0155**	39 (35 %)	37 (33 %)	0.779
Serious events	77 (62 %)	89 (75 %)	**0.0463**	73 (63 %)	94 (82 %)	**< 0.001**	64 (58 %)	75 (68 %)	0.128
Awareness	68 (55 %)	76 (64 %)	0.0796	63 (55 %)	81 (70 %)	**0.0028**	36 (32 %)	40 (36 %)	0.573
Pneumonia	66 (53 %)	78 (66 %)	**0.0477**	60 (52 %)	86 (75 %)	**0.0363**	72 (65 %)	73 (66 %)	0.889
Hyperthermia	55 (44 %)	69 (58 %)	**0.0164**	51 (44 %)	81 (70 %)	0.0571	31 (28 %)	21 (19 %)	0.114
Hair loss	102 (82 %)	100 (84 %)	0.11	97 (84 %)	92 (80 %)	0.387			
Osteoporosis	101 (81 %)	96 (81 %)	0.925	100 (87 %)	87 (82 %)	0.393			
Gastric ulcer	94 (76 %)	90 (76 %)	0.629	88 (77 %)	88 (77 %)	0.781			
Impaired smell	87 (70 %)	73 (61 %)	0.137	72 (63 %)	57 (50 %)	0.066			
Flatulence	69 (56 %)	80 (67 %)	**0.0468**	59 (51 %)	65 (57 %)	0.246			

**C**	**Effect size**	**p-value**	**Effect size**	**p-value**
Nausea	0.222	0.123	0.024	0.794
Allergic reaction	0.500	**0.003**	-0.049	0.691
Sore throat	0.216	0.123	0.217	**0.035**
Puncture injury	0.249	0.084	-0.047	0.652
Puncture infection	0.254	0.086	-0.019	0.853
Damage teeth	0.442	**0.014**	0.147	0.378
Thrombosis/embolism	0.225	0.221	0.177	0.226
Blood loss	0.171	0.333	0.151	0.309
POD	0.249	0.138	0.284	**0.030**
Hypotension	0.151	0.366	0.285	0.054
Laryngospasm	0.004	0.983	0.042	0.768
Serious events	0.091	0.593	0.173	0.238
Awareness	0.059	0.743	0.156	0.336
Pneumonia	0.348	0.051	-0.187	0.254
Hyperthermia	-0.076	0.701	0.170	0.346

Table S7. Anaesthesiologist survey. A Communication with patient. B Documents provided to anaesthesiologist by the patient during PAA visit. 4-point Likert scale: 1-I do not agree, 2- I do rather not agree, 3-I rather agree, 4-I agree.

A	Control (N=135)	Experimental (N=140)	P-Value	B	Control (N=135)	Experimental (N=140)	P-Value
**Professional experience**				**Medication plan**			
< 1 year	32 (29 %)	23 (21 %)	0.311	Yes	36 (27 %)	57 (42 %)	**0.015**
1 - 4 years	36 (33 %)	44 (41 %)		No	96 (73 %)	80 (58 %)	
> 5 years	26 (24 %)	17 (16 %)		Missing	3	3	
> 10 years	16 (15 %)	24 (22 %)					
Missing	25	32					
**New information**				**GP letter**			
1	14 (13 %)	8 (7 %)	0.798	Yes	2 (2 %)	11 (8 %)	**0.02**
2	29 (26 %)	34 (31 %)		No	130 (98 %)	126 (92 %)	
3	40 (36 %)	40 (37 %)		Missing	3	3	
4	27 (25 %)	26 (24 %)					
Missing	25	32					
**Required documents**				**Cardiologist findings**			
1	7 (6 %)	6 (6 %)	0.235	Yes	8 (6 %)	14 (10 %)	0.268
2	12 (11 %)	8 (7 %)		No	124 (94 %)	123 (90 %)	
3	22 (20 %)	18 (17 %)		Missing	3	3	
4	69 (63 %)	76 (70 %)					
Missing	25	32					
**Patient asked questions**				**Pulmonary function test**			
1	15 (14 %)	4 (4 %)	0.314	Yes	0 (0 %)	4 (3 %)	0.122
2	58 (53 %)	68 (63 %)		No	132 (100 %)	133 (97 %)	
3	32 (29 %)	29 (27 %)		Missing	3	3	
4	5 (5 %)	7 (6 %)					
Missing	25	32					
**Effective communication**				**Anaesthesia card/ allergy passport**			
1	1 (1 %)	2 (2 %)	0.179	Yes	7 (5 %)	6 (4 %)	0.782
2	2 (2 %)	4 (3 %)		No	125 (95 %)	131 (96 %)	
3	25 (23 %)	30 (28 %)		Missing	3	3	
4	82 (75 %)	72 (67 %)					
Missing	25	32					
**Need for information**				**Other doctor’s letters**			
1	33 (30 %)	25 (23 %)	0.333	Yes	16 (12 %)	20 (15 %)	0.594
2	55 (50 %)	60 (56 %)		No	116 (88 %)	117 (85 %)	
3	19 (17 %)	17 (16 %)		Missing	3	3	
4	3 (3 %)	6 (6 %)					
Missing	25	32					
**Detailed education refused**				**No documents**			
Yes	47 (43 %)	34 (31 %)	0.094	Yes	81 (61 %)	64 (47 %)	**0.02**
No	63 (57 %)	74 (69 %)		No	51 (39 %)	73 (53 %)	
Missing	25	32		Missing	3	3	

Table S8. Amsterdam Perioperative Anxiety Score (APAIS). P-values indicate differences between groups as denoted by #. For reasons of clarity only significant p-values ≤ 0.05 are shown.

	Control			Experimental			
	**Baseline**	**ICT**	**PAA**	**Baseline**	**ICT**	**PAA**	**P-value**
**Anaesthesia-related anxiety**							
**I am worried about the anaesthetic.**							
Mean (±SD)	1.85 (± 0.920) **#**	1.87 (± 0.934)	1.84 (± 0.840)	2.11 (± 1.02) **#**	2.07 (± 1.01)	1.97 (± 0.951)	**0.026**
Median [Q1, Q3]	2.00 [1.00, 2.00]	2.00 [1.00, 2.00]	2.00 [1.00, 2.00]	2.00 [1.00, 3.00]	2.00 [1.00, 2.00]	2.00 [1.00, 2.00]	
Missing	11		11	13	1	23	
**The anaesthetic is on my mind continually.**							
Mean (±SD)	1.56 (± 0.789) **#**	1.70 (± 0.865)	1.65 (± 0.798)	1.80 (± 0.992) **#**	1.88 (± 1.03)	1.78 (± 1.07)	**0.05**
Median [Q1, Q3]	1.00 [1.00, 2.00]	1.00 [1.00, 2.00]	1.00 [1.00, 2.00]	2.00 [1.00, 2.00]	2.00 [1.00, 2.00]	1.00 [1.00, 2.00]	
Missing	11		11	13	1	23	
**Surgery-related anxiety**							
**I am worried about the procedure.**							
Mean (±SD)	2.65 (± 1.14)	2.55 (± 1.07)	2.46 (± 1.15)	2.71 (± 1.11)	2.50 (± 1.08)	2.47 (± 1.11)	
Median [Q1, Q3]	3.00 [2.00, 4.00]	2.00 [2.00, 3.00]	2.00 [2.00, 3.00]	3.00 [2.00, 4.00]	2.00 [2.00, 3.00]	2.00 [2.00, 3.00]	
Missing	11		11	13	1	23	
**The procedure is on my mind continually.**							
Mean (±SD)	2.49 (± 1.17)	2.39 (± 1.07)	2.35 (± 1.11)	2.64 (± 1.20)	2.48 (± 1.18)	2.35 (± 1.18)	
Median [Q1, Q3]	2.00 [1.75, 3.25]	2.00 [2.00, 3.00]	2.00 [2.00, 3.00]	2.00 [2.00, 4.00]	2.00 [2.00, 3.00]	2.00 [1.00, 3.00]	
Missing	11		11	13	1	23	
**Demand for information**							
**I would like to know as much as possible about the anaesthetic.**							
Mean (±SD)	2.80 (± 0.963)	2.67 (± 0.999)	2.73 (± 0.931)	2.83 (± 1.03)	2.76 (± 1.09)	2.77 (± 1.16)	
Median [Q1, Q3]	3.00 [2.00, 4.00]	3.00 [2.00, 3.00]	3.00 [2.00, 4.00]	3.00 [2.00, 4.00]	3.00 [2.00, 4.00]	3.00 [2.00, 4.00]	
Missing	11		11	13	1	23	
**I would like to know as much as possible about the procedure.**							
Mean (±SD)	3.35 (± 0.988)	3.13 (± 0.996)	3.06 (± 1.16)	3.44 (± 1.01)	3.21 (± 1.09)	3.13 (± 1.10)	
Median [Q1, Q3]	4.00 [3.00, 4.00]	3.00 [2.00, 4.00]	3.00 [2.00, 4.00]	4.00 [3.00, 4.00]	3.00 [2.00, 4.00]	3.00 [2.00, 4.00]	
Missing	11		11	13	1	23	
**Total APAIS score**							
Mean (±SD)	14.7 (± 4.14)	14.3 (± 4.28)	14.1 (± 4.14)	15.5 (± 4.61)	14.9 (± 4.79)	14.5 (± 4.67)	
Median [Q1, Q3]	14.5 [6.00]	14.0 [6.00]	14.0 [6.00]	15.0 [5.25]	14.0 [5.00]	14.0 [6.00]	
Missing	11		11	13	1	24	

Table S9. Choice education mode

Anaesthesia education is mandatory. If I have the choice, I prefer…	**Control (N=124)**	**Experimental (N=119)**	**P-Value**
.. online education at home with explanatory videos and texts.	14 (11 %)	10 (9 %)	0.088
.. personal explanation by the anaesthesiologist.	41 (33 %)	26 (22 %)	
.. a combination of both.	69 (56 %)	81 (69 %)	
Missing		2	

Table S10. Patient satisfaction with PAA appointment. Rated on a 4-point Likert scale: 1-I do not agree, 2- I do rather not agree, 3-I rather agree, 4-I agree.

	Control (N=135)	Experimental (N=140)	P-Value
**The waiting time in the anesthesia outpatient clinic was reasonable.**			
1	10 (8 %)	12 (10 %)	0.235
2	16 (13 %)	24 (20 %)	
3	31 (25 %)	25 (21 %)	
4	67 (54 %)	58 (49 %)	
Missing	11	21	
**I had enough time to ask my questions about the anaesthesia.**			
1	0 (0 %)	0 (0 %)	0.746
2	0 (0 %)	0 (0 %)	
3	4 (3 %)	3 (3 %)	
4	120 (97 %)	116 (97 %)	
Missing	11	21	
**The anaesthesiologist has taken sufficient time to provide information.**			
1	0 (0 %)	0 (0 %)	0.784
2	0 (0 %)	0 (0 %)	
3	5 (4 %)	4 (3 %)	
4	119 (96 %)	115 (97 %)	
Missing	11	21	
**The anaesthesia consultation was understandable, conclusive and complete. I feel comprehensively informed and educated.**			
1	0 (0 %)	0 (0 %)	0.951
2	1 (1 %)	0 (0 %)	
3	6 (5 %)	7 (6 %)	
4	117 (94 %)	112 (94 %)	
Missing	11	21	
**Any fears or worries I may have had were completely allayed during the consultation.**			
1	1 (1 %)	0 (0 %)	0.633
2	5 (4 %)	10 (8 %)	
3	28 (23 %)	25 (21 %)	
4	90 (73 %)	84 (71 %)	
Missing	11	21	

Table S11. Satisfaction with online anaesthesia education.

	Experimental (N=140)
**Online anaesthesia education was understandable, conclusive and complete. I feel comprehensively informed and educated.**	
1	0 (0 %)
2	1 (1 %)
3	11 (8 %)
4	128 (91 %)
**The time required for online anaesthesia education was appropriate.**	
1	0 (0 %)
2	2 (1 %)
3	9 (6 %)
4	129 (92 %)
**It is good that I was able to organize my own time for the online anaesthesia consultation.**	
1	0 (0 %)
2	0 (0 %)
3	5 (4 %)
4	135 (96 %)
**Any fears or worries were completely allayed by online anaesthesia information.**	
1	3 (2 %)
2	14 (10 %)
3	65 (46 %)
4	58 (41 %)
**Any questions after digital anaesthesia information you would like to discuss with anaesthesiologist?**	
Yes	46 (33 %)
No	94 (67 %)
**How did you view the online information on anaesthesia and fill out the health questionnaire?**	
Alone	110 (96 %)
Together with others	5 (4 %)
Missing	25
**How often did you look at the online anaesthesia information?**	
Once	96 (83 %)
More than once	19 (17 %)
Missing	25
